# Construction of a *miR-15a*-based risk prediction model for vascular calcification detection in patients undergoing hemodialysis

**DOI:** 10.1080/0886022X.2024.2313175

**Published:** 2024-02-29

**Authors:** Chen Fu, Yingjie Liu, Huayu Yang, Qiaojing Liang, Wenhu Liu, Weikang Guo

**Affiliations:** a Department of Nephrology, Faculty of Kidney Diseases, Beijing Friendship Hospital, Capital Medical University, Beijing, PR China; b Division of Geriatrics, Medical and Health Care Center, Beijing Friendship Hospital, Capital Medical University, Beijing, PR China

**Keywords:** Hemodialysis, *miR-15a*, vascular calcification, abdominal aortic calcification score (AACS), nomogram model

## Abstract

Vascular calcification (VC) is highly prevalent in patients undergoing hemodialysis, and is a significant contributor to the mortality rate. Therefore, biomarkers that can accurately predict the onset of VC are urgently required. Our study aimed to investigate serum *miR-15a* levels in relation to VC and to develop a predictive model for VC in patients undergoing hemodialysis at the Beijing Friendship Hospital hemodialysis center between 1 January 2019 and 31 December 2020. The patients were categorized into two groups: VC and non-VC. Logistic regression (LR) models were used to examine the risk factors associated with VC. Additionally, we developed an *miR-15a*-based nomogram based on the results of the multivariate LR analysis. A total of 138 patients under hemodialysis were investigated (age: 58.41 ± 13.22 years; 54 males). VC occurred in 79 (57.2%) patients. Multivariate LR analysis indicated that serum *miR-15a*, age, and WBC count were independent risk factors for VC. A *miR-15a*-based nomogram was developed by incorporating the following five predictors: age, dialysis vintage, predialysis nitrogen, WBC count, and *miR-15a*. The receiver operating characteristic (ROC) curve had an area under the curve of 0.921, diagnostic threshold of 0.396, sensitivity of 0.722, and specificity of 0.932, indicating that this model had good discrimination. This study concluded that serum *miR-15a* levels, age, and white blood cell (WBC) count are independent risk factors for VC. A nomogram constructed by integrating these risk factors can be used to predict the risk of VC in patients undergoing hemodialysis.

## Introduction

Mortality rates for patients with kidney failure treated with dialysis remain unacceptably high, with annual mortality rates ranging from 15 to 20% [1]. Cardiovascular disease (CVD) is the leading cause of death among patients undergoing hemodialysis [2]. Furthermore, vascular calcification (VC) is a powerful independent risk factor for cardiac events and mortality in patients undergoing hemodialysis [3,4]. It is highly prevalent in patients with chronic kidney disease (CKD), particularly those with kidney failure [5]. The incidence of VC among patients undergoing hemodialysis ranges from 67 to 90.7% [4,6,7]. Age and prolonged vintage during chronic dialysis are established risk factors for developing VC; other features, including diabetes mellitus (DM), deregulated divalent ion balance, vitamins K and D deficiencies, and secondary hyperparathyroidism, have also been implicated in increasing the risk of uremic VC [8,9]. However, the risk factors for VC vary across studies. VC is currently identified using noninvasive imaging modalities, such as chest radiography and computed tomography (CT), or invasive techniques, such as intravascular ultrasound of the coronary arteries, which are all widely used. However, factors, such as cost, availability, and radiation exposure may limit their accessibility to some patients [8,10]. Accordingly, the identification of circulating biomarkers that can identify the presence of VC in the blood could offer a more appealing alternative [11]. However, there are currently no reliable diagnostic or prognostic biomarkers for evaluating VC, especially in patients undergoing hemodialysis.

MicroRNAs (miRNAs) are non-coding RNAs with short nucleotide sequences that participate in the post-transcriptional regulation of biological functions and play key roles in abnormal physical conditions [12]. miRNAs function as negative regulators of translation and are involved in many cellular processes [12]. Many miRNAs exist in the circulation as well as in tissues, blood, and urine [13,14]. Serum miRNAs have good stability [15], have been studied as potential biomarkers for various diseases, including kidney diseases [16], and have become biomarkers for the early diagnosis and chronic progression of kidney damage [17,18]. VC progression occurs not only because of plasma oversaturation and passive calcium deposition, but also because of active osteogenesis in the tunica media of the arterial wall [19]. Several molecules involved in bone and mineral metabolism may also play key roles in cardiovascular calcification formation [20–22]. As reported by Takaaki et al. miR-16-5p, miR-17-5p, miR-20a-5p, and miR-106b-5p target the VEGFA–VEGFR2 signaling pathway, thereby inhibiting VC induced by elevated phosphorus levels [23]. According to initial research [24], miRNAs may contribute to the development of VC by controlling phenotypic changes in vascular smooth muscle cells (VSMCs) and the balance of calcium and phosphate. In a subsequent study, we used miRNA profiles previously analyzed by our group in an *in vitro* model of human VSMC biomineralization and identified the miRNA closely associated with VC as *miR-15a* [25]. Our subsequent study also found that bone marrow mesenchymal stem cell-derived exosomes play a role in calcification inhibition by transferring *miR-15a/15b/16a* and inhibiting their common target gene, *NFATc3*, which downregulates OCN expression and thus inhibits VSMC osteogenic trans-differentiation [26]. We selected *miR-15a* as the target for further investigation. It is yet to be determined whether there is a significant difference in *miR-15a* expression in the circulation of patients undergoing hemodialysis who have VC compared to those who do not. Therefore, a group of patients undergoing hemodialysis was recruited to evaluate *miR-15a* expression.

This cross-sectional study aimed to identify the association between *miR-15a* expression and VC and determine whether *miR-15a* is a novel risk factor for VC, independent of traditional risk factors. Nomograms have recently received increasing attention and are widely used for predicting disease risk because they are intuitive and allow for visualization of data [27]. Therefore, these images were used to visualize the data analysis results. This study also aimed to develop and validate a nomogram model using miRNA biomarkers and conveniently measure factors to identify VC, thereby facilitating timely diagnoses, and treatment in clinical practice.

## Patients and methods

### Patient selection

This cross-sectional study recruited patients undergoing hemodialysis at the Beijing Friendship Hospital Hemodialysis Center between 1 January 2019 and 31 December 2020. The ethics committee of Beijing Friendship Hospital, which is affiliated with Capital Medical University, approved this study (ethics committee approval number: 2018-P2-224-01). Written informed consent was obtained from all participants or their legal proxies. The study was conducted in accordance with the principles of the Declaration of Helsinki.

**Figure 1.**
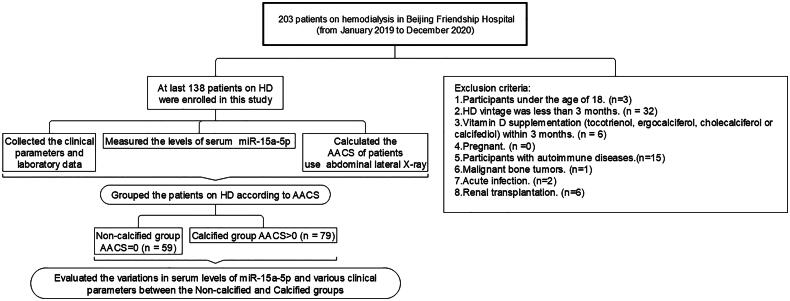
Flowchart of the cross-sectional study.

Adult patients on hemodialysis (aged 18 years or older) were eligible for study participation if they had been treated with hemodialysis for more than 3 months. The exclusion criteria were as follows: (a) vitamin D supplementation (tocotrienol, ergocalciferol, cholecalciferol, or calcifediol) within the past 3 months; (b) pregnancy; and (c) autoimmune diseases, malignant bone tumors, acute infection, and renal transplantation (Figure 1). All patients underwent dialysis three times per week. Dialysate A contained sodium chloride, potassium chloride, calcium chloride, magnesium chloride, glacial acetic acid, and an appropriate amount of dialysis water. Dialysate B contained sodium bicarbonate with an appropriate amount of dialysis water. The dialysate calcium concentration was 1.5 mmol/L, the dialysate flow rate was 500 mL/min, the blood flow rate was 200–300 mL/min, and the dialysis vintage was 4 h each time.

### Demographic, clinical, and biochemical data

Demographic and clinical data, including age, sex, dialysis vintage, and comorbidities, were obtained from participant interviews and a review of the medical records. Blood samples were collected before and after the dialysis. The following biochemical variables were measured: hemoglobin, white blood cell (WBC) count, potassium, calcium, phosphate, ferritin, creatinine, predialysis nitrogen, post-dialysis nitrogen, uric acid, intact parathyroid hormone (iPTH), albumin (bromocresol green method), and the urea reduction ratio (URR). The URR after dialysis was estimated according to the following equation: URR = ([Upre–Upost]/Upre)∗100% [28]. Laboratory measurements were performed immediately before initiating the Monday or Tuesday hemodialysis session, which was scheduled exactly 68 h after the previous session (Friday or Saturday). Blood samples were obtained from a central venous catheter, arteriovenous fistula, or graft. An additional 4 mL of EDTA-anticoagulated blood was collected from each patient prior to dialysis, which was centrifuged at 4 °C for 20 min at 3000 rpm. The centrifuged serum samples were transferred into sterile Eppendorf (EP) tubes using a sterile pipette and stored at −80 °C for subsequent quantitative detection of miRNAs.

### Imaging examinations

**Figure 2.**
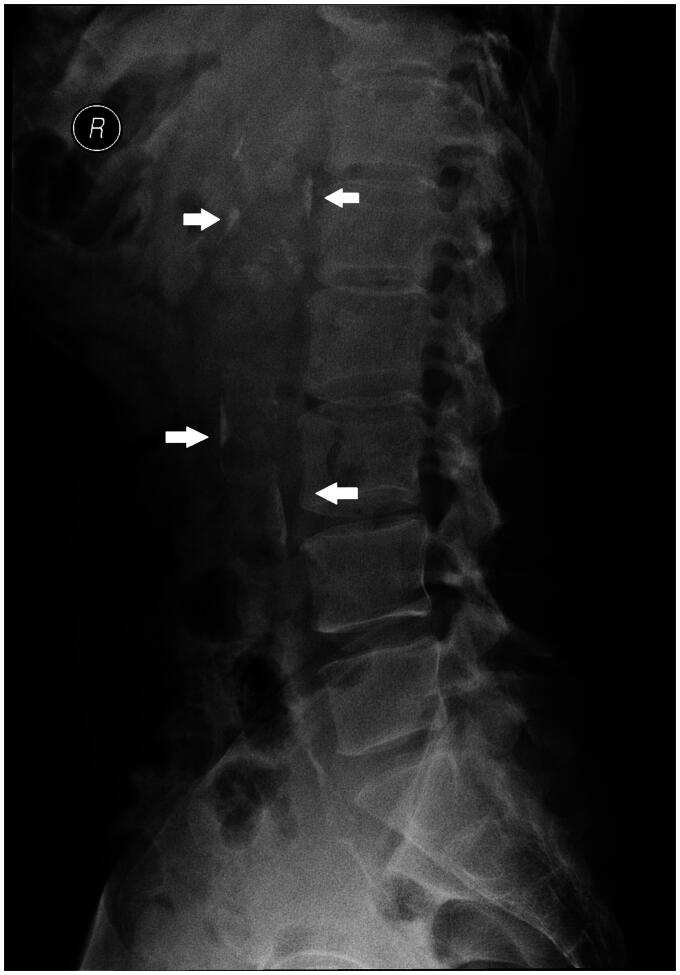
Radiological quantification of abdominal aortic calcification using abdominal lateral X-ray. Arrowhead denotes calcification sites.

The abdominal aortic calcification score (AACS) was assessed by performing abdominal lateral radiography at baseline for all patients included in the study. Based on the AACS score, participants were divided into three groups: non-calcified group (AACS = 0, *n* = 59), mild calcified group (0 < AACS ≤ 4, *n* = 44), moderate calcified group (4 < AACS ≤ 16, *n* = 31), and severe calcified group (16 < AACS ≤ 24, *n* = 4). An X-ray machine (DIAGNOST, German Philips) was used to perform abdominal lateral radiography (Figure 2), and AACS was evaluated based on the Kauppila scoring system [29]. The scoring criteria included defining the aorta within the corresponding vertebral levels of the L1–L4 segments on lateral abdominal radiographs. The extent of calcification involving the anterior and posterior walls of the aorta in each segment was scored as 1 point (calcification range < 1/3), 2 points (calcification range 1/3–2/3), or 3 points (calcification range > 2/3). The total scores for the anterior and posterior walls of the abdominal aorta in each segment were summed to obtain an AACS score (0–24 points) for each patient. All selected patients were independently evaluated by two radiologists who were blinded to their clinical data. If the difference between the two scores was > 5 points, a third radiologist evaluated the score and the average of the three scores was used as the AACS score.

### Determination of circulating microRNA levels

Cell-free total RNA, including miRNAs, was extracted from the serum using Trizol (Invitrogen Cat. No. 15596018), in accordance with the manufacturer’s instructions. Purified extracts were then reverse-transcribed with the All-in-One^™^ miRNA First-Strand cDNA Synthesis Kit version 2.0 (Gene Copoeia, Rockville, MD 20850 USA, Cat. No. QP113) and processed to quantitative PCR using the Taq Pro Universal SYBR qPCR Master Mix (Vazyme, Cat. No. Q712). Internal control samples were used in each PCR plate to reduce inter-plate variability, and all measurements were performed in triplicate. The primers used were as follows: forward primer: 5′-TAGCAGCACATAATGGTTTGTG-3′ for *miR-15a-5p*; forward primer: 5′-TCACCGGGTGTAAATCAGCTTG-3′ for *cel-miR-39-3p* standard RNA (ribobio, Cat. No. miRB0000010-3-1). The target gene fragment was identified by PCR. With *cel-miR-39-3p* standard RNA as the external reference, the quantitative results were calculated using the 2^−ΔΔCT^ method.

### Statistical analyses

Continuous variables are expressed as mean ± standard deviation (SD). Categorical variables are expressed as percentages. Univariate analyses were performed to compare differences between the two groups. The Student’s t-test was used to compare normally distributed data, while the Mann–Whitney U test was used for non-normally distributed data. Categorical data were compared using the *χ*^2^ test. The Kruskal–Wallis rank-sum test was used to compare relative miR-15a expression between groups. The Wilcoxon test was used to compare other groups with the ‘non-calcification’ group.

#### Model development

The risk factors selection process for model inclusion consisted of three steps. In the first step, a univariate logistic regression (LR) was applied to explore the nine candidate predictors with VC in the patients. In the second step, based on the magnitude of the Wald statistic of univariate analysis, variables that were statistically significant in the univariate analysis (*p* < 0.05) and applicable to real clinical scenarios were selected for inclusion in the multivariate LR analysis. Consequently, backward stepwise selection based on the criteria of Akaike Information Criterion (AIC) was applied to select final independent predictors from variables in the multivariate LR analysis to construct the prediction model. We developed an *miR-15a* nomogram based on the results of the multivariate LR analysis.

#### Model evaluation and comparison

The performance of the *miR-15a*-based model was evaluated with respect to discrimination and calibration in the training set. The area under the receiver operating characteristic (ROC) curve (AUC) was used to evaluate model discrimination. Calibration was assessed using a calibration curve along with the Hosmer–Lemeshow test to evaluate the goodness-of-fit of the model. Moreover, the clinical utility of the prediction model was evaluated by sensitivity and specificity. To prove the excellent performance of the *miR-15a*-based model, we added the comparisons of the *miR-15a*-based model, only the *miR-15a* model and only the predictors other than *miR-15a* model in discrimination and clinical utility.

All analyses were two-tailed, and *p* < 0.05 was considered to be statistically significant. The R language version 4.2.1 (R Foundation for Statistical Computing, Vienna, Austria) was used for all statistical analyses.

## Results

### Characteristics of patients undergoing hemodialysis with VC

**Figure 3.**
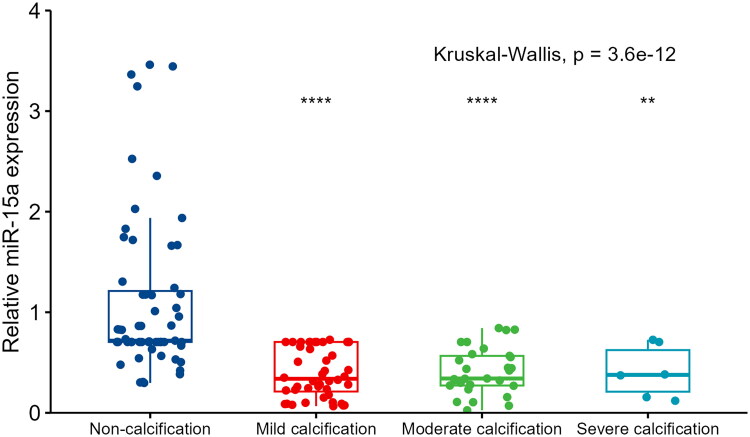
The association between serum *miR-15a* levels in patients and vascular calcification severity. The Kruskal–Wallis rank-sum test was used to compare relative miR-15a expression between groups. The Wilcoxon test was used to compare other groups with the ‘non-calcification’ group. ^****^p < 0.00001; ***p < 0.001; **p < 0.01. Comparisons between the other three groups showed no significant differences.

The mean age of patients was 58.41 ± 13.22 years, and 74 of the patients (54%) were male. The overall prevalence of VC was 57.2% (79 of 138 patients). Patients were stratified according to VC severity upon enrollment (no VC group, 42.8%; mild calcification group, 31.9%; moderate calcification group, 22.5%; and severe calcification group, 2.9%, respectively). Patients undergoing hemodialysis in the calcification group were older (*p* < 0.001), had a longer dialysis vintage (*p* = 0.002), lower predialysis nitrogen levels (*p* = 0.012), higher WBC counts (*p* = 0.015), higher calcium levels (*p* = 0.015), and lower serum *miR-15a* levels (*p* < 0.001) than patients in the non-calcification group (Table 1). Serum *miR-15a* level at baseline were significantly higher in patients without VC (1.11 ± 0.79) than those in patients with VC (0.40 ± 0.24). The difference level of *miR-15a* between the non-calcification group and the groups with each level of calcification was statistically significant; however, no difference was observed between the mild, moderate, and severe groups: no VC group 0.717, mild calcified group, 0.339; moderate calcification group, 0.341; and severe calcification group, 0.377 (Figure 3).

**Table 1.** Characteristics of patients undergoing hemodialysis with vascular calcification.

Characteristic	Overall, *N* = 138^a^	Non-calcified group, *N* = 59^a^	Calcified group, *N* = 79^a^	*p* Value^b^
Age, year	58.41 (13.22)	53.19 (13.89)	62.31 (11.28)	<0.001
Sex (male%)	74 (54%)	31 (53%)	43 (54%)	0.8
Dialysis vintage, month	110.06 (76.20)	89.85 (77.41)	125.15 (72.14)	0.002
Diabetes, %	18 (13%)	4(6.8%)	14 (17.7%)	0.059
Hypertension, %	97 (70.3%)	39 (66%)	58 (73.4%)	0.4
AACS	3.40 (5.12)	0.00 (0.00)	5.94 (5.54)	<0.001
Predialysis creatinine, mmol/L	895.81 (247.06)	946.09 (231.17)	858.25 (253.24)	0.086
Uric acid, umol/L	385.55 (70.82)	399.44 (75.68)	375.18 (65.55)	0.14
Predialysis blood urea nitrogen, umol/L	23.77 (5.03)	25.02 (5.00)	22.84 (4.89)	0.012
Hemoglobin, g/L	115.09 (12.77)	115.73 (14.06)	114.61 (11.78)	0.7
White blood cell count, *10^9^/L	6.46 (2.05)	5.98 (1.82)	6.82 (2.15)	0.015
Ferritin, ng/dL	194.62 (113.01)	176.33 (99.16)	208.28 (121.16)	0.2
Albumin, g/L	38.66 (2.85)	39.25 (2.55)	38.22 (3.00)	0.051
Potassium, mmol/L	4.88 (0.65)	4.90 (0.54)	4.87 (0.72)	0.5
Phosphate, mmol/L	1.82 (0.44)	1.83 (0.46)	1.82 (0.43)	0.9
Calcium, mmol/L	2.31 (0.22)	2.26 (0.22)	2.35 (0.22)	0.015
URR	75.82 (50.42)	70.08 (9.05)	69.75(10.55)	0.849
iPTH, pg/mL	277.91 (254.05)	233.32 (188.16)	311.22 (290.54)	0.070
miR-15a	0.70 (0.65)	1.11 (0.79)	0.40 (0.24)	<0.001

^a^ Mean (SD); *n* (%).

^b^ Wilcoxon rank sum test; Pearson’s Chi-squared test.

AACS: Abdominal aortic calcification score; URR: urea reduction ratio; iPTHintact parathyroid hormone

### Association of VC with serum miR-15a level and other variables in patients undergoing hemodialysis

Nine candidate variables, including age, dialysis vintage, predialysis creatinine, uric acid, predialysis nitrogen, WBC count, albumin, calcium, and *miR-15a* (all *p* < 0.05) were significantly associated with VC in univariate LR analyses (Table 2). Among these, five variables were included in the final prediction model. Age (*p* = 0.035), WBC count (*p* = 0.028), and *miR-15a* (*p* < 0.001) were identified as independent risk factors for VC in the subsequent multivariate regression analysis (Table 2).

**Table 2.** Univariate and multivariate logistic regression analysis of the result of vascular calcification and clinical candidate predictors.

Variables	Univariate analysis		Multivariate analysis	
	OR (95% CI)	*p*	OR (95% CI)	*p*
Age, year	1.059 (1.030–1.092)	<0.001	1.048 (1.005–1.097)	0.035
Dialysis vintage, m	1.007 (1.002–1.012)	0.008	1.006 (0.999–1.014)	0.081
Predialysis creatinine	0.998 (0.997–1.000)	0.042	–	–
Uric acid	0.995 (0.990–1.000)	0.050	–	–
Predialysis nitrogen	0.909 (0.838–0.978)	0.015	0.888 (0.775–1.002)	0.069
White blood cell count	1.243 (1.042–1.506)	0.020	1.362 (1.044–1.821)	0.028
Albumin, g/dL	0.876 (0.769–0.990)	0.038	–	–
Calcium, mg/dL	7.314 (1.474–41.014)	0.018	–	–
*miR-15a*	0.002 (0.0001–0.018)	<0.001	0.0023 (0.0002–0.0205)	<0.001

CI: confidence interval; OR: odds ratio; iPTHintact parathyroid hormone

### miR-15a-based nomogram construction and performance assessment

**Figure 4.**
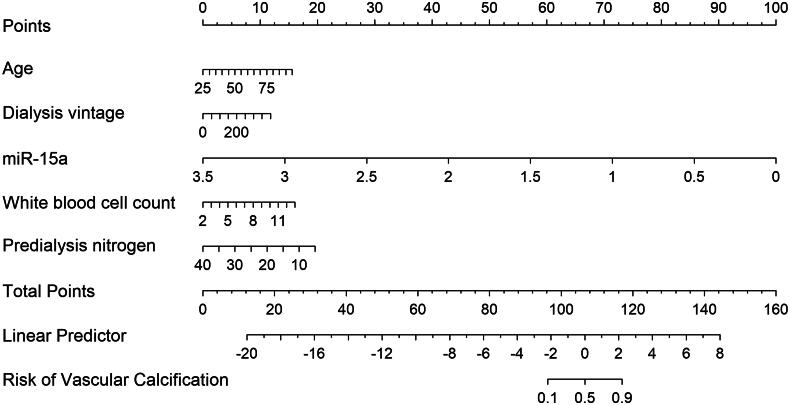
Discrimination of the *miR-15a*-based nomogram for determining vascular calcification in the patients.

**Figure 5.**
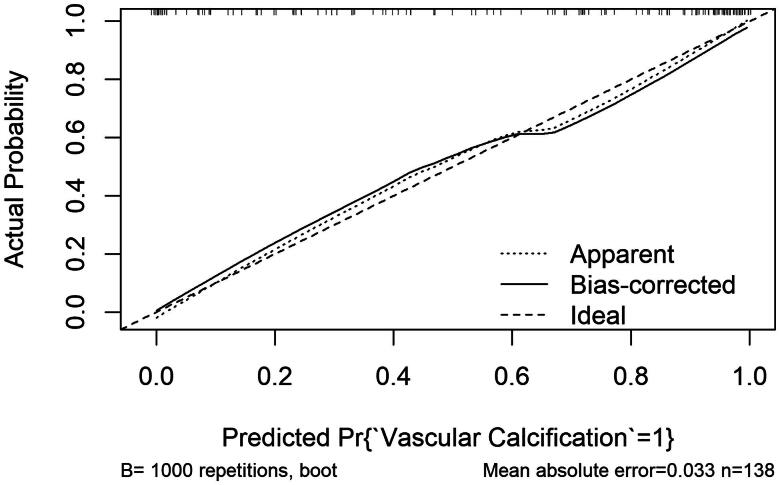
*miR-15a*-based nomogram for predicting the probability of vascular calcification in patients. The range of the total points for the nomogram is 0–160. The X-axis represents the predicted risk of VC development in patients undergoing hemodialysis. The Y-axis displays the actual CVC diagnosis. The dashed diagonal line represents the ideal prediction of an ideal model. The solid line represents the performance of the nomogram, with closer proximity to the dashed line indicating higher accuracy of the predictions of the model.

**Figure 6.**
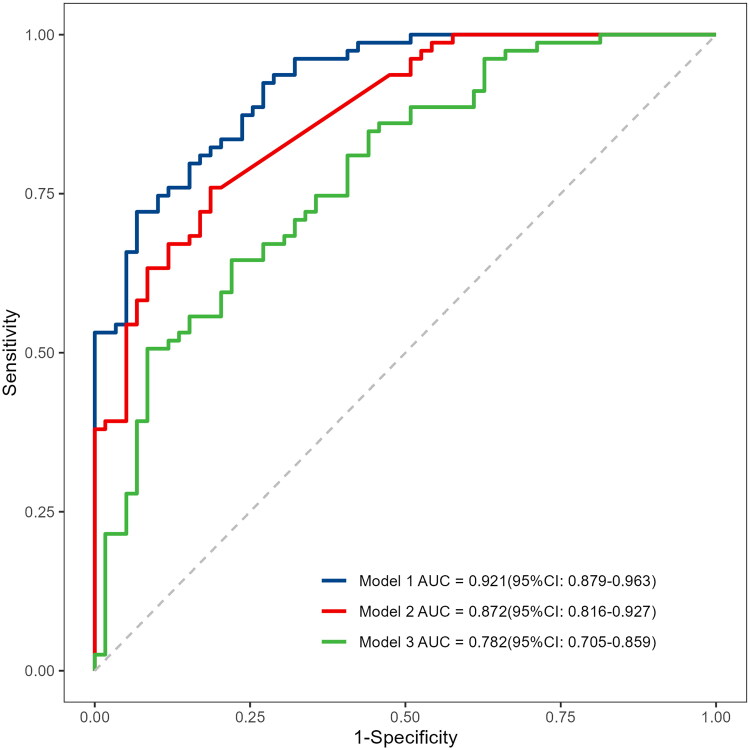
Comparison of the three models in discrimination ability. Model 1: *miR-15a*+age + dialysis vintage + predialysis nitrogen + white blood cell count, Model 2: *miR-15a*, Model 3: Age + dialysis vintage + predialysis nitrogen + white blood cell count.

A miR-15a-based nomogram was developed by incorporating the following five predictors: age, dialysis vintage, predialysis nitrogen, white blood cell count, and miR-15a (Figure 4). The calibration curve suggests good agreement between the model prediction and actual observations in the dataset (Figure 5). The Hosmer–Lemeshow test yielded a nonsignificant *p* value of 0.575, indicating good calibration power. The ROC curve of the miR-15a-based model (Model 1) had an AUC of 0.921, diagnostic threshold of 0.396, sensitivity of 0.722, and specificity of 0.932, see Supplementary material Figure S1. After 1000 bootstrap internal validations, the ROC curve showed an AUC of 0.921 (95% confidence interval [CI]: 0.879–0.963), indicating that the model had good discrimination (Figure 6, the blue curve).

The comparisons of three predictive models: the miR-15a-based model (Model 1), only the miR-15a model (Model 2), and only predictors other than the miR-15a model (Model 3) are shown in Table 3. The AUC of Model 1 is 0.921 (95% CI: 0.879–0.963), which is better than Model 2 0.872 (95% CI: 0.816–0.927, *p* = 0.008) and Model 3 0.782 (95% CI: 0.705–0.859, *p* < 0.001) (Figure 6). Moreover, Model 1 has the highest accuracy (0.812) and sensitivity (0.932) for predicting VC in patients undergoing hemodialysis.

**Table 3.** Evaluation of prediction models.

Model	Variable	Accuracy	Sensitivity	Specificity	AUC (95% CI)	*p* Value for DeLong test
Model 1	*miR*-*15a*+age + dialysis vintage + predialysis nitrogen + white blood cell count	0.812	0.932	0.722	0.921 (0.879–0.963)	Reference
Model 2	*miR-15a*	0.783	0.814	0.760	0.872 (0.816–0.927)	0.008
Model 3	age + dialysis vintage + predialysis nitrogen + white blood cell count	0.703	0.780	0.647	0.782 (0.705–0.859)	<0.001

AUC: area under receiver operator curve; CI: confidence interval

## Discussion

VC is a major cause of cardiovascular morbidity and mortality in patients with CKD [30,31]. VC is a complex process that leads to the pathological accumulation of calcium phosphate crystals in the intimal and medial layers of the vessel wall. The presence of these mineral-rich plaques leads to the hardening of arteries, which increases the likelihood of fibrosis, inflammation, and oxidative stress at the cellular level, thereby putting patients at risk [30]. From a clinical standpoint [31,32], VC has the potential to directly elevate the chances of numerous clinical complications, including the deterioration of atherosclerosis and an increased risk of vascular incidents, such as heart attack, stroke, and vascular occlusive events. Therefore, there is an urgent need to identify effective biomarkers of VC.

Based on our findings, *miR-15a* plays a crucial role in inhibiting calcification and osteogenic trans-differentiation [26]. In view of the results of the *in vitro* experiments, we performed a cross-sectional study in patients undergoing hemodialysis, in which the collected uremic sera were analyzed for *miR-15a* expression to assess its association with abdominal aortic calcification in patients under hemodialysis. We further identified some common, well-established risk factors for VC, such as age, dialysis vintage, predialysis creatinine, uric acid, predialysis nitrogen, WBC count, albumin, calcium, and *miR-15a*. Serum *miR-15a* levels, age, and WBC count were independent risk factors for VC. Serum *miR-15a* expression is a valid biomarker for assessing VC in patients undergoing hemodialysis. The prediction model included age, dialysis vintage, WBC count, the expression of *miR-15a*, and predialysis nitrogen, which successfully predicted the presence of VC in patients undergoing hemodialysis. This nomogram provides an accurate visual tool for the medical staff for prediction, early intervention, and graded management.

In this study, the prevalence of VC was 57.2%. Previous studies have reported a wide range of VC incidences among hemodialysis patients, ranging from 54.6 to 100% [4,6,7]. This higher incidence of calcification in the previous literature may be due to the inclusion of not only abdominal aortic calcification, but also thoracic aortic, coronary artery, and echocardiographic calcifications in the mitral valve, aortic valve, or mitral annulus. Furthermore, our study utilized abdominal radiography to assess VC, whereas other studies employed different imaging modalities, including coronary CT [33], plain hand [34], and abdominal [4] radiography. CT scans offer higher sensitivity for calcification than plain abdominal radiography, potentially contributing to the higher incidence of VC in some studies on patients undergoing hemodialysis than in our study. An important issue with the use of CT to evaluate VC is the exposure to ionizing radiation [35]. In our study, abdominal radiographs were used to evaluate abdominal aortic calcification because radiography is a more convenient bedside assessment tool that results in lower radiation exposure for patients. To avoid exposure to ionizing radiation, this study aimed to identify a biomarker for predicting VC.

*miR-15a* belongs to the *miR-15* family, which is associated with the pathogenesis of cancer, neurological illnesses, and CVDs [36–38]. Some miRNAs are associated with VC. Panizo et al. [39] reported that *miR-29b*, *miR-133b*, and *miR-211* play direct roles in vascular smooth muscle calcification induced by high phosphorus levels. Previous research has shown that the overexpression of *miR-15a* can reduce the osteogenic trans-differentiation and calcification of VSMCs [26]. In our study, we examined the expression of *miR-15a* in the serum and explored the possibility of using *miR-15a* as a diagnostic biomarker for VC in hemodialysis patients. We observed that the serum expression of *miR-15a* in the calcification group was significantly lower than that in the non-calcification group, and the mean *miR-15a* level in the non-calcification group was 2.78 times higher than that in the calcification group. However, in the calcified group, *miR-15a* expression did not decrease with increasing calcification severity, which may be due to the small sample size. A significant difference was observed between the mild calcification and non-calcification groups, indicating that *miR-15a* has good discriminatory ability for calcification in the early stages. Thus, *miR-15a* can be considered as an indicator of early-stage calcification. Even after adjusting for covariates, such as age and WBC count, an independent correlation between serum *miR-15a* and VC remained. These results demonstrate the critical role of *miR-15a* in VC in patients undergoing hemodialysis. ROC curve analysis was performed to evaluate the diagnostic significance of *miR-15a* in patients undergoing hemodialysis. These results indicate that *miR-15a* can serve as an indicator of VC in patients with kidney failure undergoing hemodialysis. *miR-15a*, age, predialysis nitrogen, dialysis vintage, and WBC count formed a joint prediction factor with an AUC of 0.921, exhibiting high sensitivity and specificity for predicting VC in patients undergoing hemodialysis. These results suggest that the joint detection of *miR-15a* with age, predialysis nitrogen level, dialysis vintage, and WBC count can serve as a preliminary screening method for predicting VC in patients undergoing hemodialysis, while avoiding additional radiation and costs. This may also provide new therapeutic targets for the treatment of VC. In addition, Liu et al. [40] identified *microRNA-211-5p* as a biomarker in the early detection of uremic VC among patients with kidney failure. Furthermore, Chao et al. [41,42] found that VC severity correlated with decreased serum levels of *miR-125b-2-3p* and *mir 378a-3p* and the miRNA/mRNA pair *miR-378a-30/SULF1* in combination with traditional clinical features appears to be useful for improved diagnosis and classification of the severity of uremic VC in patients with kidney failure. However, in these studies, the populations studied were not exclusively hemodialysis patients [40], and relevant prediction models were not provided [42].

In our study, age was an independent risk factor for calcification, which is consistent with previous findings [4, 6, 42]. Although existing literature lacks reports on the association between the WBC count and uremic calcification, our study demonstrated that the WBC count is also an independent risk factor for VC in patients undergoing hemodialysis and that high WBC counts increase the risk of calcification. Hou et al. [43] reported a borderline or significantly positively association of the total WBC and eosinophil counts with the probability of coronary artery atherosclerosis 20 years later; furthermore, multiple studies have shown a correlation between elevated levels of WBCs and the presence of arterial stiffness and other atherosclerotic events [44, 45]. Several biological mechanisms contribute to the onset and exacerbation of inflammation and oxidative stress, including mitochondrial activity, xanthine oxidase, and Nicotinamide Adenine Dinucleotide Phosphate (NADPH) oxidase, which exacerbate VC in patients with CKD [19]. Because activated WBCs can adhere to the vascular endothelium, penetrate the intima, induce capillary leukostasis, increase vascular resistance, and release hydrolytic enzymes, cytokines, and growth factors, it is widely accepted that chronic low-grade inflammation in the arterial wall may have a critical role in both the initiation and progression of CVDs. These factors released by activated WBCs have the potential to induce further vascular damage [46]. Phosphate metabolism disorders are important triggers of CKD mineral bone disease [21, 47]. Previous studies have suggested that blood phosphorus is a factor that leads to VC progression during hemodialysis [6, 30, 42]. However, in our study, the blood phosphorus level was not found to be a risk factor for calcification, which may be because this dialysis center adheres to the use of phosphate binders, resulting in minimal changes in serum phosphate levels during data collection. Other studies arrived at similar conclusions [21, 42].

As far as we know, there have been no studies examining the usefulness of serum *miR-15a* in predicting the likelihood of VC. This is the first nomogram of miRNA participation constructed to predict VC in patients undergoing hemodialysis. Although the prognostic nomogram in this study showed a good predictive ability, some limitations should be considered. First, the associations between dialysis vintage and albumin levels were not statistically significant, possibly due to the small sample size. Second, as our study was retrospective, certain patient data were inevitably missing. This might have decreased the number of eligible cases. The cross-sectional study design could not determine causality, and biochemical indices were only collected at a single time point; thus, we were unable to monitor fluctuations in biochemical indices. Third, we did not measure vitamin D levels of the participants. Fourth, our findings will be more reliable if the nomogram model is externally validated using another independent large-scale dataset to verify whether our results are universally applicable.

Future research should include combining multiple miRNAs or other biomarkers with miRNAs and clinical data to establish superior models for risk prediction. Further investigations into the underlying biological mechanisms of miRNA-regulated VC may provide new insights into potential therapeutic targets for the prevention and treatment of this disease. In addition, studies exploring the potential of miRNA-based therapies, particularly those targeting *miR-15a*, may pave the way for the development of novel therapeutic interventions.

## Supplementary Material

Supplemental Material

## Data Availability

The original contributions of this study are available from the corresponding author upon reasonable request.
