# Supplementary figures and images for: Construction of a miR-15a-based risk prediction model for vascular calcification detection in patients undergoing hemodialysis
Source: Ren Fail. 2024 Feb 29;46(1):2313175. doi: 10.1080/0886022X.2024.2313175 (PMC10906117; doi:10.1080/0886022X.2024.2313175)

## S1.The calibration curve of the prediction model.

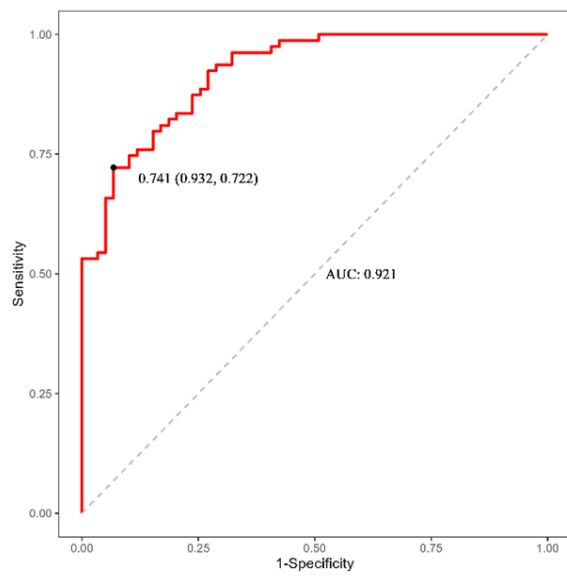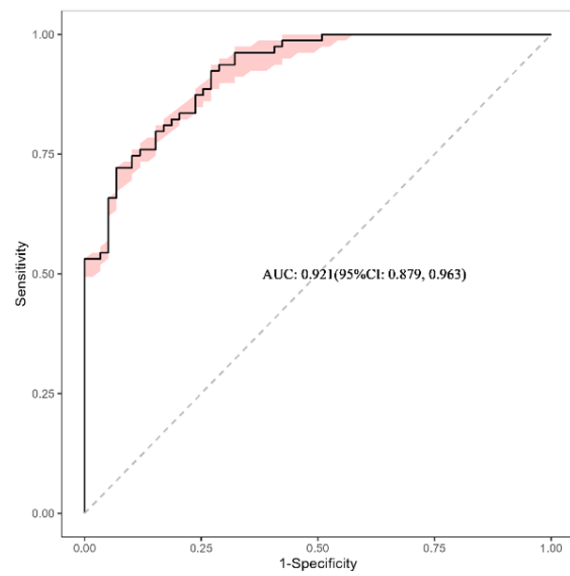

Supplement: Supplemental Material [file IRNF_A_2313175_SM1435.pdf]
